# GSK3β Inhibition Prevents Macrophage Reprogramming by High-Dose Methotrexate

**DOI:** 10.1159/000526622

**Published:** 2022-11-14

**Authors:** Israel Ríos, Baltasar López-Navarro, Mónica Torres-Torresano, Blanca Soler Palacios, Miriam Simón-Fuentes, Ángeles Domínguez-Soto, Ittai B. Muller, Gerrit Jansen, Ángel L. Corbí, Amaya Puig-Kröger

**Affiliations:** ^a^Unidad de Inmunometabolismo e Inflamación, Instituto de Investigación Sanitaria Gregorio Marañón, Hospital General Universitario Gregorio Marañón, Madrid, Spain; ^b^Departamento de Inmunología y Oncología, Centro Nacional de Biotecnología/Consejo Superior de Investigaciones Científicas, Madrid, Spain; ^c^Myeloid Cell Laboratory, Centro de Investigaciones Biológicas, Madrid, Spain; ^d^Department of Clinical Chemistry, Amsterdam University Medical Center, Amsterdam, The Netherlands; ^e^Department of Rheumatology and Clinical Immunology, Amsterdam University Medical Center, Amsterdam, The Netherlands

**Keywords:** Methotrexate, Macrophages, Macrophage reprogramming

## Abstract

Methotrexate (MTX) is an antifolate drug used as a chemotherapeutic agent for acute lymphoblastic leukemia, where MTX improves patients' prognosis. Macrophage reprogramming is being increasingly assessed as an antitumor therapeutic strategy. However, and although MTX limits the pathogenic action of macrophages in chronic inflammatory diseases, its effects on tumor-promoting macrophages have not been previously explored. We now report that MTX shapes the transcriptional and functional profile of M-CSF-dependent human macrophages, whose transcriptome is highly enriched in the gene signature that defines pathogenic tumor-associated macrophages (“large TAM”). Specifically, MTX prompted the acquisition of the gene signature of antitumoral “small TAM” and skewed macrophages toward an IL-6^high^ IFNβ1^high^ IL-10^low^ phenotype upon subsequent stimulation. Mechanistically, the MTX-induced macrophage reprogramming effect correlated with a reduction of the M-CSF receptor CSF1R expression and function, as well as a diminished expression of MAF and MAFB transcription factors, primary determinants of pro-tumoral macrophages whose transcriptional activity is dependent on GSK3β. Indeed, the ability of MTX to transcriptionally reprogram macrophages toward an antitumoral phenotype was abrogated by inhibition of GSK3β. Globally, our results establish MTX as a macrophage reprogramming drug and indicate that its ability to modulate macrophage polarization may also underlie its therapeutic benefits. Since GSK3β inhibition abrogates the reprogramming action of MTX, our results suggest that the GSK3β-MAFB/MAF axis constitutes a target for the macrophage-centered antitumor strategies.

## Introduction

Tissue macrophages exhibit a huge functional plasticity, and they can exert proinflammatory or anti-inflammatory/resolving functions depending on their ontogeny and the prevailing extracellular cues [1, 2]. Regarding ontogeny, tissue-resident macrophages mostly perform a homeostatic role and reparative and anti-inflammatory functions during inflammation, whereas newly recruited macrophages usually display proinflammatory functions [1, 3, 4, 5, 6]. Best exemplifying the influence of the environment on macrophage functional polarization, GM-CSF primes macrophages (GM-MØ) to acquire robust immunostimulatory and proinflammatory activity, whereas M-CSF prompts macrophages (M-MØ) to gain an anti-inflammatory and immunosuppressive (IL-10^high^ TNF^low^ IL-6^low^) profile [7]. In line with their effector functions, M-MØ and GM-MØ exhibit distinct transcriptional profiles that resemble those of tumor-associated macrophages (TAM) and rheumatoid arthritis synovial macrophages in vivo [8, 9], respectively.

In the case of cancer, macrophages are involved in the initiation and progression of the tumor. TAM normally associate to bad prognosis as they are “educated” by tumor-derived factors (e.g., TGF-β, VEGF, M-CSF, IL-10) to exhibit anti-inflammatory, immunosuppressive, and pro-tumoral functions, lastly contributing to tumor progression, metastasis, and even resistance to chemotherapy, radiotherapy, and immunotherapy [2, 10]. Consequently, metabolic and functional reprogramming of macrophages is considered a promising therapeutic strategy for cancer and other inflammatory disorders, where deregulated polarization has a pathogenic role [10, 11, 12]. TAM reprogramming aims at converting immunosuppressive pro-tumoral macrophages into immunostimulatory and antitumor effector cells, and targeting various Toll-like receptors (TLR), the M-CSF/CSF1R axis, and its downstream effector PI3Kγ have been already assayed to that end in clinical trials for cancer therapy [11, 13]. Indeed, blockade of M-CSF/CSF1R leads to macrophage reprogramming [14, 15] and loss of TAM in the tumor [16], while blockade of macrophage PI3Kγ in an animal model of pancreatic ductal adenocarcinoma reprograms TAM for immunostimulation and inhibition of tumor metastasis [17, 18].

One-carbon metabolism, mediated by the folate cofactor, enables cancer growth and proliferation by supporting pyrimidine and purine biosynthesis, as well as amino acid homeostasis, epigenetic maintenance, and redox defense [19]. Inhibition of folate metabolism and/or nucleotide biosynthesis is an important anticancer strategy. The main antifolates in current clinical practice are methotrexate (MTX), which primarily targets dihydrofolate reductase and pemetrexed, which mostly targets thymidylate synthase [20]. MTX preferentially target rapidly dividing cells and is an important component of chemotherapy for breast cancer, head and neck cancer, non-Hodgkin lymphoma, osteosarcoma, bladder cancer, choriocarcinoma and for children with acute lymphoblastic leukemia (ALL), where high-dose MTX can significantly increase cure rates and improve patients' prognosis [21, 22, 23, 24]. However, the role of MTX in myeloid cells within the tumor microenvironment has not been previously explored.

We now report that MTX reprograms human M-CSF-primed monocyte-derived macrophages (M-MØ) at the transcriptional and functional level, and that the MTX reprogramming effect is dependent on GSK3β. Mechanistically, MTX downregulates the expression of CSF1R and MAF/MAFB transcription factors and, consequently, impairs the acquisition of the MAF-dependent pro-tumoral phenotype of M-MØ. Our results establish MTX as a macrophage reprogramming drug and evidence that its therapeutic benefits go beyond limiting tumor cell proliferation, suggesting that GSK3β might be the final target of the antitumor strategies, aiming at CSF1R or PI3Kγ.

## Methods

### Cell Culture

Human peripheral blood mononuclear cells (PBMCs) were isolated from buffy coats from normal donors over a Lymphoprep (Nycomed Pharma) gradient. Monocytes were purified from PBMC by magnetic cell sorting using CD14 microbeads (Miltenyi Biotech). Monocytes were cultured at 0.5 × 10^6^ cells/mL for 7 days in RPMI 1640 (standard RPMI, which contains 1 mg/L folic acid) supplemented with 10% fetal calf serum, at 37°C in a humidified atmosphere with 5% CO_2_, and containing GM-CSF (1,000 U/mL; ImmunoTools) to generate GM-CSF-polarized macrophages (GM-MØ) or M-CSF (20 ng/mL; ImmunoTools) to generate M-CSF-polarized macrophages (M-MØ). GM-CSF or M-CSF was added every 2 days. MTX (MTX; Pfizer) and pemetrexed (PMX; Sigma-Aldrich) were used at 5 µM on monocytes at the beginning of the differentiation procedure together with M-CSF or in 5-day M-CSF-differentiated macrophages. Where indicated, folinic acid (FA; Sigma-Aldrich) was used at 500 µM. MTX was dissolved in PBS, whereas PMX and FA were initially dissolved in H_2_O and later in RPMI. Whenever MTX, PMX, or FA were used, control experiments were done by exposing macrophages to the same amount of solvent. LPS (10 ng/mL, 0111:B4 strain; InvivoGen), 2′3′cGAMP (10 µg/mL; InvivoGen) was added at the indicated time points onto 7-day fully differentiated macrophages.

### RNAseq and GSEA

Total RNA was isolated from three independent preparations and processed at BGI (https://www.bgitechsolutions.com), where library preparation, fragmentation, and sequencing were performed using the BGISEQ-500 platform. An average of 5.41 Gb bases was generated per sample and, after filtering, clean reads were mapped to the reference (UCSC Genome assembly hg38) using Bowtie2 (average mapping ratio 93.41%). Gene expression levels were calculated by using the RSEM software package. Differential gene expression was assessed by using DEseq2 algorithms using the parameters fold change >2 and adjusted *p* value <0.05. For gene set enrichment analysis (GSEA) (http://software.broadinstitute.org.gsea/index.jsp), the gene sets available at the website, as well as the “M-MØ-specific LPS-induced” and “GM-MØ-specific LPS-induced” gene sets (GSE99056), the M-MØ-specific” and “GM-MØ-specific” gene sets (GSE188278), and the MAF and MAFB regulates genes (GSE155719). The gene signatures of Large Tumor-Associated macrophages (“Large TAM”) and “Small TAM” from colorectal liver metastasis were derived from GSE131353 [25]. “GM-MØ-specific” and “M-MØ-specific” gene sets include all the genes whose expression is significantly different in GM-MØ and M-MØ (adjusted *p* <0.05 and log_2_ FC > 3). The “GM-MØ-specific” gene set includes 430 genes, and the “M-MØ-specific” gene set contains 216 genes (GSE188278). The data reported in this publication have been deposited in NCBIs Gene Expression Omnibus and are accessible through GEO Series accession number GSE186151 (MTX-treated M-MØ), GSE189740 (LPS activated MTX-treated M-MØ), GSE185872 (CHIR-99021-treated M-MØ), and GSE188278 (monocyte-to-macrophage differentiation).

### Quantitative Real-Time RT-PCR

Total RNA was retrotranscribed, and cDNA was quantified using the Universal Human Probe Roche library (Roche Diagnostics). Quantitative real-time PCR (qRT-PCR) was performed on a LightCycler® 480 (Roche Diagnostics). Assays were made in triplicates and results normalized according to the expression levels of TBP. Results were obtained using the ΔΔ^CT^ method for quantitation. The oligonucleotides used to quantify mRNA transcripts were (5′-3′): GDF15 forward: ccggatactcacgccaga; GDF15 reverse: agagatacgcaggtgcaggt; INHBA forward: ctcggagatcatcacgtttg; INHBA reverse: ccttggaaatctcgaagtgc; IL1β forward: ctgtcctgcgtgttgaaaga; IL1β reverse: ttgggtaatttttgggatctaca; LIF forward: tgccaatgccctctttattc; LIF reverse: gtccaggttgttggggaac; TBP forward: cggctgtttaacttcgcttc; TBP reverse: cacacgccaagaaacagtga.

### ELISA

Supernatants from M-MØ were tested for the presence of IL-6, IL-10 (BioLegend), and IFNβ, CCL3, CCL4, and CCL8 (R&D Systems) following the procedures supplied by the manufacturers.

### Western-Blot

Cell lysates were obtained in RIPA buffer containing 1 mM PIC (Protease Inhibitor Cocktail; Sigma), 10 mM NaF, 1 mM Na_3_VO_4,_ and 0.5 mM DTT. Ten to thirty micrograms cell lysate was subjected to SDS-PAGE and transferred onto an Immobilon polyvinylidene difluoride membrane (Millipore). For folate receptor beta (FOLR2, FRβ), cell lysates were subjected to SDS-PAGE under nonreduced conditions. Protein detection was carried out using rabbit polyclonal antibodies against pp38, pJNK, and pERK (clones D3F9, 81E11 and D13.14.4E; Cell Signaling, 1/1,000), pIRF3 (clone 4D4G; Cell Signaling, 1/1,000), pSTING (clone D7C3S; Cell Signaling, 1/1,000), STING (clone D2P2F; Cell Signaling, 1/1,000), pCSF1R (clone 49C10; Cell Signaling, 1/1,000), CD209 (dsg-1, 1/1,000) [26], MAF (sc-7866; Santa Cruz Biotech, 1/1,000), MAFB (clone O91E9; BioLegend, 1/1,000), and pSTAT3 (clone D3A7; Cell Signaling, 1/2,000), goat polyclonal against CSF1R (AF329; R&D Systems, 1/2,000), and mouse monoclonal antibody against human FOLR2 (FRβ, kindly provided by Dr. Takami Matsuyama [27], 1/800). Protein loading was normalized using an antibody against GAPDH (clone 6C5; Santa Cruz Biotechnology, 1/2,000) or against human vinculin or tubulin (clone VIN-11-5, 6-11B-1; Sigma-Aldrich, 1/3,000).

### Mixed Leukocyte Reaction

Five-day M-CSF-differentiated macrophages were exposed to 5 µM MTX for 48 h. M-MØ and MTX-M-MØ (d5) were detached using PBS with 2 mM EDTA at 37°C, and replated in 96-well U-bottom plates (10^4^ cells/well) in RPMI with 5% human AB serum (Lonza). Allogeneic T-lymphocytes were isolated from PBMCs using CD3+ magnetic beads (Miltenyi Biotec) and co-cultured with macrophages at 1:10 M-MØ:T lymphocyte ratio for 4 days in RPMI with 5% human AB serum. Then, ^3^H-Thymidine (1 µCi/well; Perkin Elmer) was added and, after 16 h, radioactivity was transferred to a filter and thymidine counts measured in a scintillation counter (Perkin Elmer).

### Statistical Analysis

Statistical analysis was done using GraphPad Prism, using parametric Student's *t* test, as appropriate, and one-way ANOVA test coupled with Tukey's post hoc test were indicated. Two-sided *p* value <0.05 was considered significant (*p < 0.05; **p < 0.01, ***p < 0.001).

## Results

### MTX Promotes Monocyte Differentiation into Macrophages with a Proinflammatory Transcriptional and Functional Profile

**Fig. 1:**
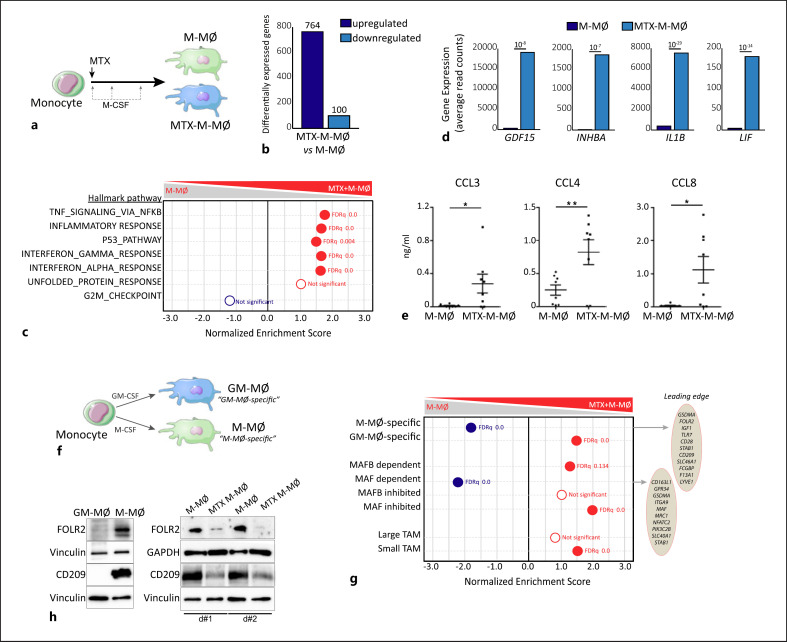
High-dose MTX promotes a proinflammatory gene expression profile in M-CSF monocyte-derived macrophages. **a** Schematic representation of the experiments. Monocytes were untreated or exposed to 5 µM MTX at the beginning of the 7-day macrophage differentiation process with M-CSF, and the RNA levels were determined at day 7 on M-MØ and MTX-M-MØ. **b** Number of annotated genes whose expression is upregulated or downregulated in M-MØ after 7d of MTX treatment (adjusted *p* <0.05). **c** Summary of GSEA with the indicated gene set on the ranked comparison of the transcriptomes of M-MØ versus MTX-M-MØ, using the Hallmarks v7.2 data set available at the website. False discovery rate (FDRq) is indicated (red, positive enrichment; blue, negative enrichment). The genes within the leading edge of each GSEA are indicated in online supplementary Table 1 (see www.karger.com/doi/10.1159/000526622 for all online suppl. material). **d** Relative level of expression of the indicated genes as determined by RNA-sequencing on M-MØ and MTX-M-MØ (GSE186151). **e** Production of CCL3, CCL4, and CCL8 by M-MØ and MTX-M-MØ (d5). Mean ± SEM of 8 independent donors are shown (*p < 0.05, **p < 0.01, paired *t* test). **f** Schematic representation of the experiment. Monocytes were exposed to GM-CSF or M-CSF during the monocyte-to-macrophage differentiation process, and RNA levels were determined at day 7 on GM-MØ and M-MØ. The “GM-MØ-specific markers” and “M-MØ-specific markers” data set are available at GEO (https://www.ncbi.nlm.nih.gov/geo/) (GSE188278). **g** Summary of GSEA of the genes significantly modulated by GM-CSF (GM-MØ-specific markers) and M-CSF (M-MØ-specific markers), the top 100 MAFB and MAF-regulated genes in M-MØ (GSE155719) and the “small TAM” and “large TAM” signatures (GSE131353) on the ranked comparison of M-MØ versus MTX-M-MØ transcriptomes. Informative genes found in the leading edge and FDRq are indicated (red, positive enrichment; blue, negative enrichment). **h** Immunoblot analysis of FOLR2 (FRβ) and CD209 by monocytes differentiated with GM-CSF (GM-MØ) versus M-CSF (M-MØ) (left) and MTX-M-MØ versus M-MØ (right, two independent donors are shown). Vinculin and GAPDH protein levels were determined as protein loading controls.

We have previously demonstrated that low-dose MTX enhances the proinflammatory nature of GM-CSF-dependent monocyte-derived macrophages (GM-MØ) via a p53/TS axis, whereas it has minimal effect on M-CSF-dependent macrophages (M-MØ) [28]. Since high-dose MTX is commonly used in cancer therapy [29, 30], we sought to determine the gene expression profile of M-MØ generated in the continuous presence of 5 µM MTX (Fig. 1a), a concentration achieved in the plasma of ALL patients subjected to high-dose MTX therapy [31, 32]. RNAseq of the resultant cells revealed that MTX triggers an important transcriptional change as MTX-M-MØ exhibited significantly (|logFC| > 1; adjusted *p* <0.05) enhanced expression of 764 genes and reduced expression of 100 genes (Fig. 1b). In line with our previous findings [28], MTX-M-MØ were enriched in genes within the GSEA Hallmark “p53 PATHWAY” term and inflammatory responses, and showed a positive enrichment of “Interferon_alpha_response” and “Interferon_gamma_response” gene sets (Fig. 1c). Besides, MTX-M-MØ had augmented expression of MTX-response genes like *GDF15, INHBA,* and *LIF* (Fig. 1d), whose upregulation was completely prevented by an excess of FA (online suppl. Fig. 1A; see www.karger.com/doi/10.1159/000526622 for all online suppl. material), demonstrating the specificity of the responses. Besides, and in agreement with the Enrichment of the GSEA Hallmark “INFLAMMATORY RESPONSE” term (Fig. 1c), MTX-M-MØ showed a higher release of the monocyte-recruitment chemokines CCL3, CCL4, and CCL8 (Fig. 1e; online suppl. Fig. 1B). Further GSEA using “GM-MØ-specific” or “M-MØ-specific” gene sets (Fig. 1f) revealed that MTX-M-MØ are positively enriched in “GM-MØ-specific” genes and also show a very significant reduction in the expression of paradigmatic “M-MØ-specific” genes like *FOLR2, CD28, IGF1*, and *CD209* (Fig. 1g). Indeed, MTX-M-MØ displayed much lower expression of FOLR2 and CD209 proteins than untreated M-MØ (Fig. 1h), supporting the relevance of the transcriptional changes induced by MTX on macrophages. Thus, exposure to high-dose MTX drives monocytes to differentiate into macrophages with reduced expression of M-MØ-specific genes and higher expression of the genes that define proinflammatory GM-MØ.

**Fig. 2:**
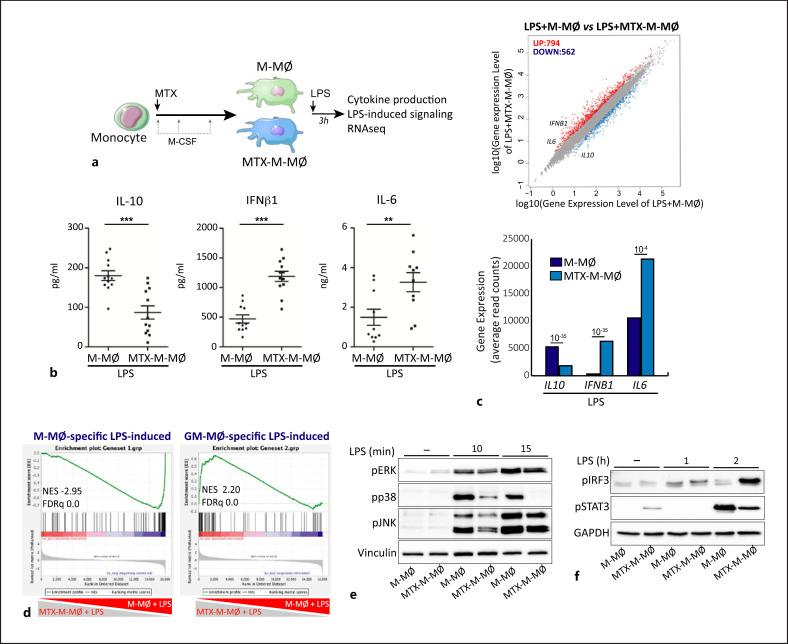
LPS activated MTX-treated M-MØ exhibit a functional proinflammatory profile. **a** Experimental design. Monocytes were untreated or exposed to 5 µM MTX at the beginning of the 7-day macrophage differentiation process with M-CSF and challenged with LPS (10 ng/mL) on day 7. Cells were assayed 3 h post-LPS stimulation on M-MØ and MTX-M-MØ. **b** Production of IL-10, IFNβ, and IL-6 by M-MØ and MTX-M-MØ challenged with LPS for 3 h as determined by ELISA. Mean ± SEM of 10–12 independent donors, each symbol represents a single donor (**p < 0.01, ***p < 0.001, paired *t* test). **c** Upper panel, scatter plot of RNAseq results showing gene expression changes 3 h post-LPS stimulation in MTX-M-MØ (LPS + MTX-M-MØ/LPS + M-MØ). The number of annotated genes whose expression is upregulated or downregulated 3 h post-LPS stimulation in M-MØ after 7 days of MTX treatment (adjusted *p* <0.05) is shown. Lower panel, relative level of expression of the indicated genes as determined by RNAseq on LPS + M-MØ and LPS + MTX-M-MØ, adjusted *p* value is indicated. **d** GSEA on the ranked comparison of the transcriptome of LPS + MTX-M-MØ versus LPS + M-MØ, using the genes significantly modulated by LPS in GM-MØ (GM-MØ-specific LPS-induced) and M-MØ (M-MØ-specific LPS-induced) as data set. The genes within the leading edge of each GSEA are indicated in online supplementary Table 1. **e, f** Immunoblot analysis of pERK, pJNK, and pp38 (**e**), pIRF3 and pSTAT3 (**f**) by monocytes differentiated with M-CSF in the absence or presence of MTX for 7 days and challenged with LPS for the indicated time points. Vinculin or GAPDH protein levels were determined as protein loading control.

The functional consequences of the continuous exposure to MTX were next assessed through the determination of the cytokine profile of MTX-M-MØ upon exposure to pathogen-associated or danger-associated molecular stimuli. Regarding pathogenic stimuli (Fig. 2a), LPS-treated MTX-M-MØ produced higher levels of IL-6 and IFNβ1, but lower levels of IL-10, than M-MØ (Fig. 2b). Moreover, the comparison of the transcriptomes of LPS-treated M-MØ and LPS-treated MTX-M-MØ (Fig. 2c) confirmed the stronger proinflammatory nature of MTX-M-MØ (lower *IL-10* and higher *IL-6* and *IFNB1* expression after LPS exposure) (Fig. 2c, lower panel). A similar conclusion was reached through GSEA using the recently described “M-MØ-specific LPS-induced” and “GM-MØ-specific LPS-induced” gene sets (GSE99056) [33] since LPS-treated MTX-M-MØ showed a positive enrichment of the latter but a negative enrichment of the “M-MØ-specific LPS-induced” gene set (Fig. 2d). In line with these findings, and compared to untreated M-MØ, LPS induced lower levels of phosphorylation of ERK, JNK, p38 MAPK, and STAT3 (Fig. 2e, f), but higher IRF3 activation (Fig. 2f) in MTX-M-MØ, implying that MTX also affects LPS-initiated intracellular signaling in M-MØ. Altogether, these results indicate that MTX conditions monocytes to differentiate into macrophages with a proinflammatory transcriptional and functional profile.

**Fig. 3:**
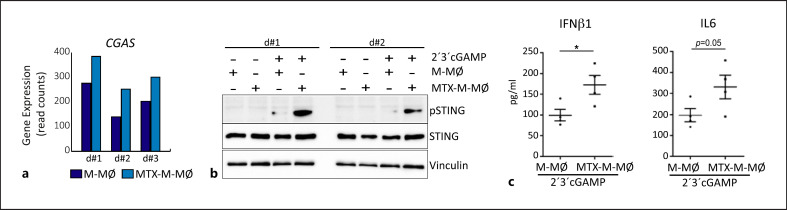
cGAMP activated MTX-treated M-MØ exhibit higher functional proinflammatory profile than M-MØ. **a** Relative level of expression of *CGAS* as determined by RNA-sequencing on M-MØ and MTX-M-MØ (GSE186151). **b** Immunoblot analysis of pSTING by monocytes differentiated with M-CSF in the absence or presence of MTX for 7 days and challenged with 2′3′cGAMP for 2 h. The medium was replaced for folic acid-free RPMI with 10% FCS for the final 16 h. Vinculin protein levels were determined as protein loading control. **c** Production of IFNβ and IL-6 by M-MØ and MTX-M-MØ challenged with 2′3′cGAMP for 7 h. Mean ± SEM of 4 independent donors are shown (*p < 0.05, paired *t* test).

In the case of danger-associated stimuli, and since the expression of *CGAS* (that codes for cGAMP synthase) was higher in MTX-M-MØ compared to untreated M-MØ (Fig. 3a), we explored the effect of MTX on the cGAS-cGAMP-STING pathway, which detects tumor-derived DNA and generates antitumor immunity via the TBK1-IRF3-dependent production of IFNβ and the NFκB pathway [34]. To that end, macrophages were exposed to cGAMP, and the phosphorylation of STING and the cytokine profile was determined. Compared to untreated M-MØ, cGAMP induced higher STING activation (Fig. 3b) and higher levels of IL-6 and IFNβ1 in MTX-M-MØ (Fig. 3c), thus indicating that MTX also alters macrophages-responses to a danger-associated stimulus commonly present in the tumor microenvironment.

To determine the extent of the influence of MTX on the M-CSF-driven differentiation of M-MØ, we also evaluated the effect of MTX on the expression of genes whose up or downregulation along monocyte differentiation is specifically promoted by either M-CSF or GM-CSF (online suppl. Fig. 2A). GSEA results indicated that MTX-M-MØ are very significantly enriched in genes exclusively upregulated along monocyte-to-GM-MØ differentiation (“upregulated Mo-to-GM-MØ-specific,” online suppl. Fig. 2B, C), while they show a negative enrichment of genes exclusively upregulated along monocyte-to-M-MØ differentiation (“Upregulated Mo-to-M-MØ-specific,” online suppl. Fig. 2B, C). Furthermore, the exact opposite results were obtained, when GSEA was done on “downregulated Mo-to-GM-MØ-specific” and on “downregulated Mo-to-M-MØ-specific” gene sets (online suppl. Fig. 2B, C). Therefore, and combined with the above findings, our results demonstrate that MTX modifies the transcriptional changes that take place along M-CSF-driven monocyte-to-macrophage differentiation.

### Altered Expression of MAF, MAFB, and CSF1R upon M-MØ Differentiation in the Presence of MTX

**Fig. 4:**
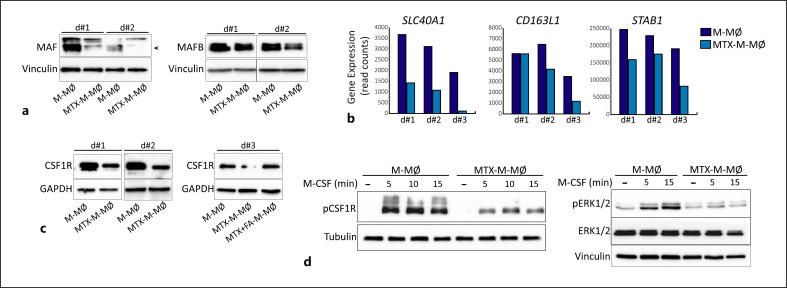
MTX modulates the expression of MAF, MAFB, and CSF1R in M-CSF monocyte-derived macrophages. **a** Immunoblot analysis of MAF and MAFB by M-MØ and MTX-M-MØ. **b** Relative level of expression of the indicated genes as determined by RNA-sequencing on M-MØ and MTX-M-MØ (GSE186151). **c** Left, Immunoblot analysis of CSF1R by M-MØ and MTX-M-MØ. Right, immunoblot analysis of CSF1R by M-MØ, MTX-M-MØ, or MTX-M-MØ exposed to FA. GAPDH protein levels were determined as protein loading control. **d** Immunoblot analysis of pCSF1R (left), pERK1/2, and total ERK (right) by M-MØ and MTX-M-MØ challenged with M-CSF for the indicated time points. For **a, c, d**, two–three independent donors are shown. Vinculin, tubulin, or GAPDH protein levels were determined as protein loading control. Arrowheads indicate the protein of interest.

The M-CSF-driven differentiation of M-MØ is dependent on CSF1R as well as on the transcription factors MAF and MAFB [35, 36, 37, 38]. In fact, Maf controls the expression of *Csf1r* in mouse macrophages, where it serves as a switch for the generation of tumor growth-promoting macrophages [39]. In line with their altered transcriptome, MTX-M-MØ exhibited lower expression of both MAF and MAFB proteins (Fig. 4a). In fact, the expression of MAF-dependent genes like *CD163L1, SLC40A1*, and *STAB1* [40] (GSE155719) was lower in MTX-M-MØ compared to untreated M-MØ (Fig. 1g, 4b). Of note, expression of the *CSF1R*-encoded M-CSF receptor was also lower in MTX-M-MØ compared to untreated M-MØ, a decreased that was abrogated by FA (Fig. 4c). In line with the lower CSF1R expression, M-CSF stimulation of MTX-M-MØ resulted in diminished phosphorylation of CSF1R and impaired ERK activation (Fig. 4d). Therefore, MTX provokes monocytes to differentiate into macrophages with reduced expression of the factors that determine the acquisition of their pro-tumoral profile, namely, MAF, MAFB, and CSF1R.

### MTX Downregulates the Expression of MAF, MAFB, and CSF1R in M-CSF-Dependent Macrophages

**Fig. 5:**
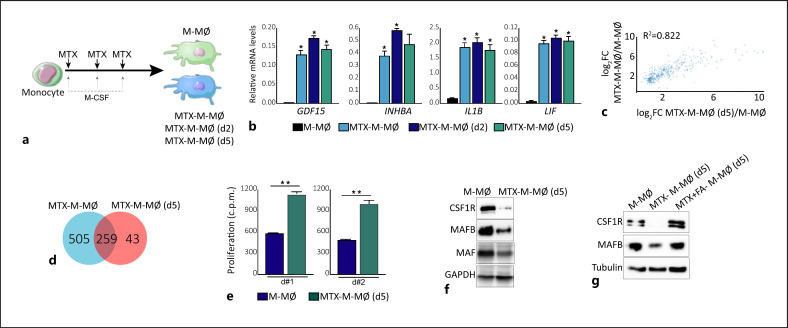
MTX modulates the expression of MAF, MAFB, and CSF1R in M-MØ. **a** Schematic representation of the experiment. Monocytes were differentiated to macrophages in the presence of M-CSF for 7 days. Five micromolar MTX was added once on monocytes [MTX-M-MØ], or on 2 days (MTX-M-MØ [d2]) or 5 days (MTX-M-MØ [d5]) after the beginning of the differentiation process with M-CSF. RNA or protein levels were determined at day 7. **b** Relative mRNA expression levels of the indicated genes as determined by qRT-PCR on M-MØ, MTX-M-MØ, MTX-M-MØ (d2), and MTX-M-MØ (d5). Mean ± SEM of three independent donors are shown. Groups were compared by applying one-way ANOVA (with Tukey's post hoc test, *p < 0.05). **c** Scatter plot of RNAseq results showing upregulated expression gene changes in MTX-M-MØ versus MTX-M-MØ (d5). **d** Venn diagram comparing the genes differentially expressed by MTX in MTX-M-MØ with the genes significantly altered by MTX in and MTX-M-MØ (d5). **e** Allogeneic CD3^+^ T-lymphocyte proliferation promoted by M-MØ and MTX-M-MØ (d5). Shown are two experiments using independent preparations of M-MØ. Mean ± SEM of six replicates performed in each experiment are shown (**p < 0.01). **f** Immunoblot analysis of CSF1R, MAFB, and MAF by M-MØ and MTX-M-MØ (d5). GAPDH protein levels were determined as protein loading control. **g** Immunoblot analysis of CSF1R and MAFB by M-MØ, MTX-M-MØ (d5), or MTX-M-MØ (d5) exposed to FA. Tubulin protein levels were determined as protein loading control.

**Fig. 6:**
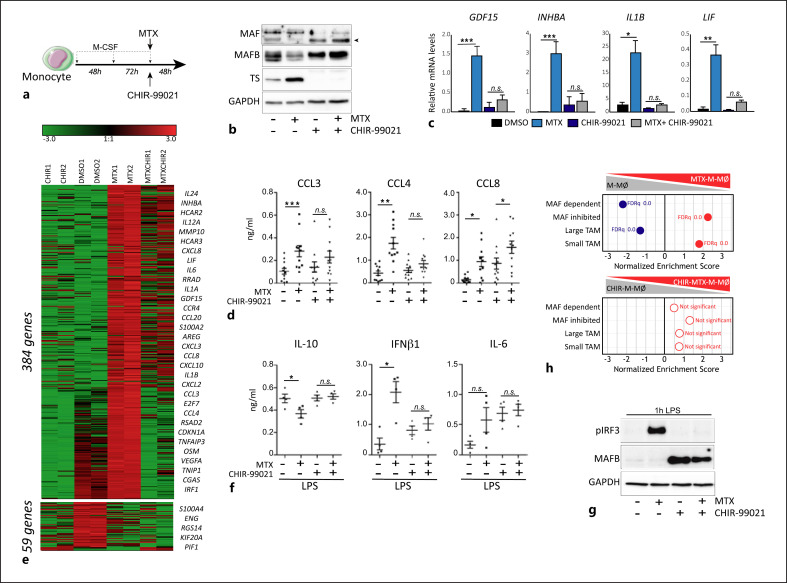
GSK3β inhibition prevents the reprogramming ability of MTX on M-MØ. **a** Experimental design. Monocytes were differentiated to M-MØ with M-CSF. On day 5, cells were untreated (DMSO, vehicle) or exposed to 5 µM MTX, 10 µM CHIR-99021 or both, and the RNA and protein levels were determined at day 7. **b** Immunoblot analysis of MAF, MAFB, and TS by M-MØ and MTX-M-MØ (d5) unexposed or exposed to CHIR-99021. GAPDH protein levels were determined as protein loading control. Arrowheads indicate the protein of interest. **c** Gene expression of the indicated genes determined by qRT-PCR on M-MØ and MTX-M-MØ (d5). MTX and CHIR-99021 were added on day 5 and gene expression determined at day 7. Mean ± SEM of 5 independent donors are shown. Groups were compared by applying one-way ANOVA (with Tukey's post hoc test, *p < 0.05, **p < 0.01, ***p < 0.001). **d** Production of CCL3, CCL4, and CCL8 by M-MØ and MTX-M-MØ (d5) unexposed or exposed to CHIR-99021. Mean ± SEM of 11 independent donors are shown. Groups were compared by applying one-way ANOVA (with Tukey's post hoc test, *p < 0.05, **p < 0.01, ***p < 0.001). **e** Heatmap of the expression of genes significantly (|logFC| > 1; *p* < 0.05) altered by MTX in the absence or in the presence of CHIR-99021 as determined by RNAseq and using Genesis (https://genome.tugraz.at/genesisclient/genesisclient_description.shtml). **f** Production of IL-10, IFNβ, and IL-6 by M-MØ and MTX-M-MØ (d5) unexposed or exposed to CHIR-99021 and challenged with LPS for 3 h. Mean ± SEM of 4 independent donors are shown. Groups were compared by applying one-way ANOVA (with Tukey's post hoc test, *p < 0.05). **g** Immunoblot analysis of pIRF3 and MAFB by M-MØ and MTX-M-MØ (d5) challenged with LPS for 1 h. GAPDH protein levels were determined as protein loading control. **h** Summary of GSEA of the top 100 MAF-regulated genes in M-MØ (GSE155719) and the “small TAM” and “large TAM” signatures (GSE131353) on the ranked comparison of M-MØ versus MTX-M-MØ (d5) transcriptomes (upper panel) and CHIR-99021-treated M-MØ versus CHIR-99021-treated MTX-M-MØ (d5) transcriptomes (lower panel). FDRq is indicated (red, positive enrichment; blue, negative enrichment). The genes within the leading edge of each GSEA are indicated in online supplementary Table 1.

Once we had demonstrated that MTX modulates the monocyte-to-macrophage differentiation, we decided to assess whether MTX could also alter the phenotype and function of differentiating macrophages. To that end, MTX was added at later time points along the monocyte-to-M-MØ differentiation process (at days 2 or 5) (Fig. 5a), and the resulting cells [MTX-M-MØ (d2) and MTX-M-MØ (d5)] were compared to M-MØ generated from MTX-treated monocytes (MTX-M-MØ). A similar upregulation of the MTX-dependent genes *GDF15, INHBA, IL1B,* and *LIF* was seen after exposure to MTX at the three time points (Fig. 5b), indicating that MTX also affects the transcriptome of differentiating M-MØ. In fact, comparison of the whole transcriptome of MTX-M-MØ and MTX-M-MØ (d5) revealed a strong overlapping of the genes whose expression is significantly upregulated in MTX-M-MØ and MTX-M-MØ (d5), as well as a positive correlation between their respective level of upregulation (*R*^2^ = 0.822) (Fig. 5c, d). At the functional level, LPS-treated MTX-M-MØ (d5) produced higher levels of IFNβ1 and lower levels of IL-10 than M-MØ (see Fig. 6f). We also assessed the T-cell stimulatory capability of MTX-M-MØ (d5) because TAMs are usually immunosuppressive [2, 41]. MTX-M-MØ (d5) showed an allostimulatory capacity that was 50% higher than the T-cell response elicited by control M-MØ (Fig. 5e). More importantly, MTX-M-MØ (d5) showed diminished MAF and MAFB protein levels as well as reduced expression of CSF1R (Fig. 5f). Like in the case of monocytes (Fig. 4c), the specificity of the MTX-induced loss of CSF1R and MAFB in MTX-M-MØ (d5) was completely abrogated by FA (Fig. 5g). Therefore, a 48 h exposure to high-dose MTX also reprograms macrophages toward the loss of MAF, MAFB, and CSF1R, and the subsequent acquisition of a more proinflammatory and immunogenic profile.

### GSK3β Inhibition Prevents the Reprogramming Ability of MTX on Human Macrophages

The protein levels and activity of the transcription factors of the “large MAF” family (MAF, MAFA, MAFB, NRL) are dependent on the phosphorylation of their transcriptional activation domains by GSK3β [42]. In fact, mutations affecting the GSK3β phosphorylation sites of “large MAF” factors result in pathologies like Aymé-Gripp syndrome (MAF), Multicentric Carpotarsal Osteolysis (MAFB), and Retinitis Pigmentosa (NRL) [42, 43]. Thus, we next assessed whether GSK3β had a role on the MTX macrophage reprogramming ability. To that end, we generated MTX-M-MØ (d5) both in the presence and absence of the GSK3β inhibitor CHIR-99021 [44], which was added immediately before MTX (Fig. 6a). CHIR-99021 pretreatment prevented the downregulation of MAF, MAFB (Fig. 6b) and the upregulation of TS (Fig. 6b) in MTX-M-MØ (d5). Besides, the GSK3β inhibitor abrogated the upregulation of MTX-responsive genes *GDF15, INHBA, LIF*, and *IL1B* in MTX-M-MØ (d5) (Fig. 6c) and the chemokines CCL3 and CCL4 (Fig. 6d). In fact, comparison of the global gene expression profile of MTX-M-MØ (d5) with or without CHIR-99021 evidenced that CHIR-99021 abolished all the significant (|logFC| > 1; adjusted *p* <0.05) MTX-induced transcriptional changes (384 upregulated, 59 downregulated) observed in MTX-M-MØ (d5) (Fig. 6e). Therefore, GSK3β inhibition impairs the macrophage transcriptional reprogramming caused by MTX. At the functional level, CHIR-99021 pretreatment reverted the lower production of IL-10 observed in MTX-M-MØ (d5) (Fig. 6f), as well as their higher production of IFNβ1 (Fig. 6f), thus implying that GSK3β inhibition also abrogates the functional reprogramming of human macrophages by MTX. Moreover, and regarding intracellular signaling, inhibition of GSK3β blocked the LPS-induced IRF3 activation in MTX-M-MØ (d5) (Fig. 6g). Altogether, these results demonstrate that the transcriptional and functional reprogramming capacity of MTX on M-CSF-primed macrophages can be fine-tuned through modulation of GSK3β activity.

Apart from MTX, pemetrexed (PMX) is another antifolate used as chemotherapeutics in mesothelioma and non-small cell lung cancer [45]. To assess whether the MTX reprogramming action can be also extrapolated to other clinically relevant antifolates, the effect of PMX on the transcriptional and functional state of human macrophages was analyzed. Like in the case of MTX, PMX upregulated MTX-dependent genes (online suppl. Fig. 3A) and downregulated the expression of MAFB in a GSK3β-dependent manner (online suppl. Fig. 3B). Additionally, PMX reduced IL-10 expression and increased IFNβ1 release from differentiating M-MØ after LPS activation (online suppl. Fig. 3C). Hence, the transcriptional and functional reprogramming action of antifolates on macrophages can be prevented by inhibition of GSK3β.

Finally, gene ontology analysis provided additional support for these experimental results. On the one hand, GSEA showed that GSK3β inhibition prevents the modulation of genes positively and negatively regulated by MAF in MTX-M-MØ (d5) (Fig. 6h), indicating that a correlation exists between the expression of MAF-regulated genes and the GSK3β-dependent macrophage reprogramming ability of MTX. On the other hand, GSEA indicated that MTX-M-MØ showed a significant enrichment of the gene set that defines the transcriptome of “small TAM,” whose presence correlates with good prognosis [25] (Fig. 1g). A similar trend was observed in MTX-M-MØ (d5) (Fig. 6h, upper panel). However, CHIR-99021 pretreatment abrogated such a positive enrichment of the “small TAM”-specific gene set (Fig. 6h, lower panel). This result is remarkable because it implies that MTX is capable of skewing the polarization of human macrophages toward the antitumoral side, and that the MTX-induced upregulation of the “small TAM”-specific gene set can be abrogated through inhibition of GSK3β. Therefore, the antitumoral effects of MTX might not be limited to its antiproliferative action on tumor cells but may also result from its ability to “reeducate” macrophages, an effect dependent on the GSK3β-MAF axis.

## Discussion

We have previously shown that low-dose MTX promotes a “trained” pro-tolerant state in GM-CSF-primed macrophages through TS- and p53-dependent mechanisms [28, 46]. Given this effect of MTX, we hypothesized that high-dose MTX, used as an antiproliferative drug in various types of cancer [20, 24], might also influence the differentiation and polarization state of anti-inflammatory and immunosuppressive monocyte-derived macrophages. We now report that MTX reprograms human M-CSF-primed monocyte-derived macrophages, whose transcriptional and functional profiles resembles that of pro-tumoral TAMs, and that MTX reprogramming can be abrogated though inhibition of GSK3β, the kinase that controls the stability and transcriptional activity of both MAF and MAFB [42]. Mechanistically, MTX downregulates the expression of CSF1R and the MAF transcription factor and, consequently, impairs the MAFB/MAF-dependent pro-tumoral phenotype of M-MØ and favors the acquisition of a proinflammatory cytokine profile. Thus, our results establish that the therapeutic benefits of MTX go beyond limiting tumor cell proliferation and include a robust macrophage reprogramming ability. It is also worth noting that MTX is capable of reprogramming monocyte-derived macrophages, which significantly contribute to the pool of macrophages present in tumors, produce factors that enable most of the hallmarks of cancer [41], and are also pathogenic in inflammatory diseases [47]. Since tissue-resident and monocyte-derived macrophages play distinct functional roles during inflammatory responses [3], it would be of interest to determine whether MTX might also affect the polarization state of tissue-resident macrophages.

Tumor-infiltrating macrophages have a critical role in tumor cell growth and metastasis, immunosuppression, and treatment resistance [2, 48]. In fact, TAM associate with poor prognosis in numerous types of tumors [2, 49], as they inhibit the generation of effective antitumor immune responses. Consequently, the elimination of the immunosuppressive activity of TAM through macrophage reprogramming is a major goal of novel therapeutic strategies in cancer [11, 49, 50, 51, 52, 53, 54]. Currently, clinical assays are underway to evaluate the validity of abrogating TAM immunosuppressive activity via macrophage reprogramming strategies [11, 49, 50, 51, 52, 55]. Among them, drugs affecting the M-CSF/CSF1R axis and PI3Kγ are being tested in advanced solid tumors, glioblastoma, sarcoma, breast cancer, and renal cell carcinoma [11, 49, 56]. The involvement of GSK3β and MAFB/MAF in the ability of MTX to re-program macrophages toward the proinflammatory and immunostimulatory side agrees with these two approaches, because M-CSF and PI3Kγ negatively regulate the GSK3β activity [42, 57]. Specifically, M-CSF binding to CSF1R leads to PI3Kγ activation [57], which ends up augmenting the inhibitory phosphorylation of GSK3 and, consequently, in higher stability and protein levels of MAF and MAFB [42]. Therefore, the ability of MTX to inhibit CSF1R and MAFB/MAF protein levels implies that MTX tackles a critical axis (CSF1R − PI3Kγ − GSK3β − MAFB/MAF) for the acquisition of the immunosuppressive and anti-inflammatory profile of human macrophages. By analogy with the action of MTX, it is tempting to speculate that GSK3β activity might be also the final target of current antitumor strategies focused on CSF1R or PI3Kγ. Besides, it might be worthy to assess whether other antifolates used as antitumor drugs (pemetrexed [PMX]) also display the macrophage reprogramming ability that we now report for MTX.

The effect of MTX on the expression of MAF is of particular interest because MAF appears to be directly associated to the acquisition of the immunosuppressive phenotype of TAM [39, 58] and also regulates Il-10 expression in mouse macrophages [59]. Studies on animal models of lung cancer have now shown that MAF is highly expressed in TAM, where it controls the immunosuppressive polarization and function and the expression of the *Csf1r* gene [39]. In fact, murine Maf downregulation results in enhanced antitumor immunity, and human MAF has been detected in tumor-infiltrating macrophages/monocytes and circulating monocytes from non-small cell lung carcinoma (NSCLC) patients [39]. In addition, mouse Maf positive regulates the expression of Il-10 [59], the paradigmatic anti-inflammatory cytokine that contributes to establish an anti-inflammatory and immunosuppressive environment in the tumor stroma. Although human IL-10 expression is more dependent on MAFB than on MAF (Simon-Fuentes et al. [33]), the reduced production of IL-10 by LPS-stimulated MTX-M-MØ concurs with the reduced expression of MAF and MAFB in MTX-treated macrophages, thus indicating that IL-10 might be the common link between the anti-inflammatory and the reprogramming ability of MTX.

The pathological relevance of the reprogramming effect of MTX on human macrophages is supported by gene ontology analysis on the transcriptional signature of TAM. The analysis of TAM from colorectal liver metastasis has identified two morphologically distinct TAM subsets (“large TAM” and “small TAM”) which exhibit distinct gene profiles and whose presence has prognostic significance [25]. Remarkably, the transcriptome of “large TAM” is very significantly enriched in M-MØ-specific genes [60, 61], whereas “small TAM” show an over-representation of the genes that define the GM-MØ-specific gene profile [60, 61] (data not shown). Extensive GSEA has revealed that MTX also favors the acquisition of genes that specifically define the “small TAM” transcriptome, and that this effect is impaired by a GSK3β inhibitor. Therefore, the GSK3β-dependent macrophage reprogramming ability of MTX could be also taken into consideration when designing combined therapeutic strategies to target myeloid cells in the tumor stroma. In this regard, PMX, another antifolate commonly used for therapy in NSCLC has recently being shown to modulate innate immune pathways in an animal model of NSCLC [62]. This previous result agrees with our finding on the ability of PMX to reprogram human differentiating macrophages.

The main limitation of our study is that the analysis of MTX effects has been restricted to in vitro generated monocyte-derived macrophages. Whereas, the molecular mechanisms we describe are clearly initiated by MTX and are dependent on MAFB/MAF-driven differentiation, we cannot rule out the possibility that additional mechanisms might be operative in vivo. That is why we are currently addressing the study of the effects of MTX on ex vivo isolated macrophages, and we are currently engaged in analyzing its actions on pathogenic tumor-associated macrophages from patients receiving MTX for therapeutic purposes as well as on a clinical assay (EudraCT number 2017-002902-11) to assess the in vivo effects of MTX on the differentiation capability of human monocytes. As a whole, our results indicate that MTX reprograms human M-CSF-primed monocyte-derived macrophages at the transcriptional and functional level in a GSK3β-dependent manner and imply that the GSK3β-MAFB/MAF axis constitutes a target for macrophage-centered antitumor strategies. Besides, our results also suggest that the macrophage reprogramming ability of MTX may contribute to its therapeutic benefits in leukemia and rheumatoid arthritis, an issue that deserves further investigation.

## Statement of Ethics

Research was conducted ethically in accordance with the World Medical Association Declaration of Helsinki. Ethical permission was approved by the Ethical Committee at Hospital General Universitario Gregorio Marañón (Report number 01/2020). Informed written consent was obtained from each human subject.

## Conflict of Interest Statement

The authors declare no potential conflicts of interest.

## Funding Sources

This work was supported by Grant PI17/00037 and PI20/00316 from Instituto de Salud Carlos III to A.P.K., Grant PID2020-114323RB-I00 from Ministerio de Ciencia e Innovación to A.L.C., “Ayudas FUNDACIÓN BBVA a equipos de investigación científica SARS-CoV-2 y COVID-19” to A.L.C., and Red de Investigación en Enfermedades Reumáticas (RIER, RD16/0012/0007), and cofinanced by the European Regional Development Fund “A way to achieve Europe” (ERDF), to A.L.C. and A.P.K. This research work was funded by the European Commission-NextGenerationEU, through CSICs Global Health Platform (PTI Salud Global). This work was supported in part by a grant from the Dutch Society for Clinical Chemistry (NVKC) to I.B. Muller and R. de Jonge. M.S.F. was funded by a Formación de Personal Investigador predoctoral fellowship from Ministerio de Ciencia e Innovación (Grant PRE2018-083396).

## Author Contributions

Israel Ríos, Baltasar López-Navarro, Mónica Torres-Torresano, Blanca Soler Palacios, Miriam Simón-Fuentes, Ángeles Domínguez-Soto, and Ittai B. Muller performed research and analyzed data; Gerrit Jansen, Ángel L. Corbí, and Amaya Puig-Kröger designed the research and analyzed the data; Ángel L. Corbí and Amaya Puig-Kröger wrote the paper.

## Data Availability Statement

The data set supporting the conclusions of this article is available in the Gene Expression Omnibus repository (http://www.ncbi.nlm.nih.gov/geo/) under accession number GSE186151, GSE188278, GSE189740, and GSE185872.

## Supplementary Material

Supplementary data

Supplementary data

Supplementary data

Supplementary data

Supplementary data
