# Supplementary material for: GSK3β Inhibition Prevents Macrophage Reprogramming by High-Dose Methotrexate
Source: J Innate Immun. 2022 Nov 14;15(1):283–96. doi: 10.1159/000526622 (PMC10643894; doi:10.1159/000526622)
Supplement: Supplementary file 2 — Supplementary data [file jin-0015-0283-s02.pdf]

Submission Declaration

By submitting this Manuscript for consideration for publication, the Author(s) agrees (agree) to adhere to S. Karger AG's Editorial and Peer Review policies, outlined below and available in full on the journal webpage. Should the Manuscript be transferred, with your agreement, to another S. Karger AG journal, this declaration remains valid. The present statement does not dictate any copyrights or licensing agreements; this will only be defined should the Manuscript be accepted for publication. During the review process, all rights related to the Manuscript remain with the Author(s).

The Author(s) declares (declare), that they agree to the following:

- The author(s) is (are) required to disclose any relationship that could reasonably be perceived by a reader as a potential conflict of interest at the time of submission. All forms of support and financial involvement (e.g. employment, consultancies, honoraria, stock ownership and options, expert testimony, grants or patents received or pending, royalties) which took place in the previous three years should be listed, regardless of their potential relevance to the paper. Also, the nonfinancial relationships (personal, political, or professional) that may potentially influence the writing of the Manuscript should be declared. If there is no conflict of interest, please state "The Author(s) declare(s) no conflict of interest".
- Financial support for the study as well as any role of funding bodies or others in study design; collection, analysis, and interpretation of data; writing of the report; any restrictions regarding the submission of the report for publication should be declared in the "Funding Sources" section of the Manuscript. If the funder had no role in any of the above, this should be clearly stated in the Manuscript's funding section.
- All individuals who have made a significant contribution to the paper are listed as Author(s) in the Manuscript and the full and correct name, affiliation and e-mail address of all Author(s) has been entered into the submission system. All the listed Author(s) has (have) received a final version of the Manuscript, take responsibility for the content and agree to its submission to, and publication in, the journal, including the order of Author(s) listed on the Manuscript.
- The work is original and has not been previously published, whole or in part, in any language unless so noted in the cover letter and with appropriate reference in the Manuscript. The consideration of translated works for publication is at the discretion of the Editor and should be declared in the cover letter and Manuscript. By submitting your Manuscript to this journal, you accept that your Manuscript may be screened for plagiarism against previously published work. For the purposes of this agreement, preprint versions (a draft before peer review) of the work that may have been shared publicly do not constitute prior publication but must be referenced in the text of your Manuscript.
- The material is not currently, and will not be, under simultaneous consideration for publication elsewhere while under consideration at this journal.
- Studies involving human subjects (including research on identifiable human material and data) must have been performed with the approval of an appropriate ethics committee and with appropriate participants' informed consent in compliance with the Helsinki Declaration.
- Experimental research on vertebrates or any regulated invertebrates must have been approved by the Author(s)' Institutional Animal Care and Use Committee (IACUC) or equivalent ethics committee and must follow internationally recognized guidelines such as the ARRIVE guidelines.
- The author(s) is (are) encouraged to make all datasets on which the conclusions of the paper rely available to editors, reviewers and readers without unnecessary restriction wherever possible. In cases where

research data are not publicly available on legal or ethical grounds, this should be clearly stated in the Data Availability Statement along with any conditions for accessing the data.

- If the Manuscript contains third-party copyright material(s), it is the Author(s)' sole responsibility to obtain permission from the relevant copyright holder for reusing the material(s), including any associated licensing fee.
- Acceptance is at S. Karger AG's discretion in accordance with its rules and processes. At any point prior to publication, including after provisional acceptance, if the Manuscript is found to be in breach of our editorial policies the Manuscript may be rejected, including rescinding acceptance. There is no prior right to publication. Manuscripts submitted will be subjected to ethical and technical checks and a peer-review process in accordance with the journal's peer review policy.
- By agreeing to participate in the peer-review process all individuals agree not to transmit or otherwise make available any information, materials or other content that is offensive; or that infringes another's rights, including any intellectual property rights.
- By agreeing to participate in the peer-review process all individuals agree not to impersonate any person or entity or falsely state or otherwise misrepresent your affiliation with a person or entity; or obtain, collect, store or modify personal information about other users.
- By agreeing to this declaration the Author(s) confirm that information given in this Manuscript submission is truthful and correct.
- The Author(s) acknowledge that Manuscripts accepted for publication by S. Karger AG will only undergo basic proofreading to check for obvious spelling and grammar mistakes. I confirm that a more in-depth language editing has been addressed prior to submission, at the Author's, respectively Authors', discretion.

A **Submitting Author** ensures that all **Co-Authors** confirm that the Submitting Author has authority to act on their behalf via the **verification link** sent out to all Co-Authors upon completing the submission.

Firmado por PUIG KRÖGER AMAYA ROCIO - ***8566** el día 06/08/2022 con un certificado emitido por AC FNMT Usuarios
