# Supplementary material for: GSK3β Inhibition Prevents Macrophage Reprogramming by High-Dose Methotrexate
Source: J Innate Immun. 2022 Nov 14;15(1):283–96. doi: 10.1159/000526622 (PMC10643894; doi:10.1159/000526622)
Supplement: Supplementary file 3 — Supplementary data [file jin-0015-0283-s03.pdf]

Supplementary Figure 1

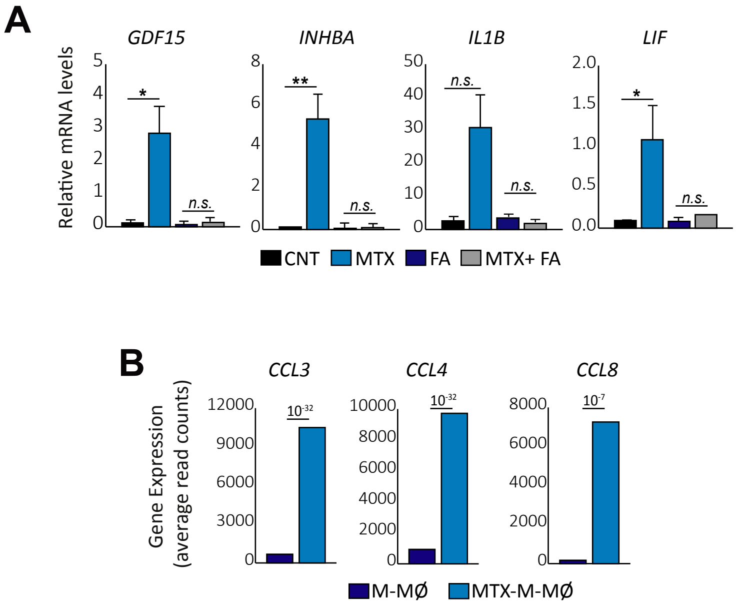

Supplementary Figure 1.- Folinic acid (FA) modulates the expression of MTX-response genes in MTX treated-M-MØ. (A) Gene expression of the indicated genes determined by qRT-PCR. Monocytes were untreated (CNT), exposed to MTX (5uM), folinic acid (500 uM) or MTX+FA at the beginning of the 7-day macrophage differentiation process with M-CSF and the RNA levels were determined at day 7 on M-MØ. Mean ± SEM of 3 independent donors are shown. Groups were compared by applying one-way ANOVA (with Tukey´s post hoc test, *p<0.05, **p<0.01). **(B)** Relative level of expression of the indicated genes as determined by RNA-sequencing on M-MØ and MTX-M-MØ (GSE186151).
