# Supplementary material for: GSK3β Inhibition Prevents Macrophage Reprogramming by High-Dose Methotrexate
Source: J Innate Immun. 2022 Nov 14;15(1):283–96. doi: 10.1159/000526622 (PMC10643894; doi:10.1159/000526622)
Supplement: Supplementary file 4 — Supplementary data [file jin-0015-0283-s04.pdf]

Supplementary Figure 2

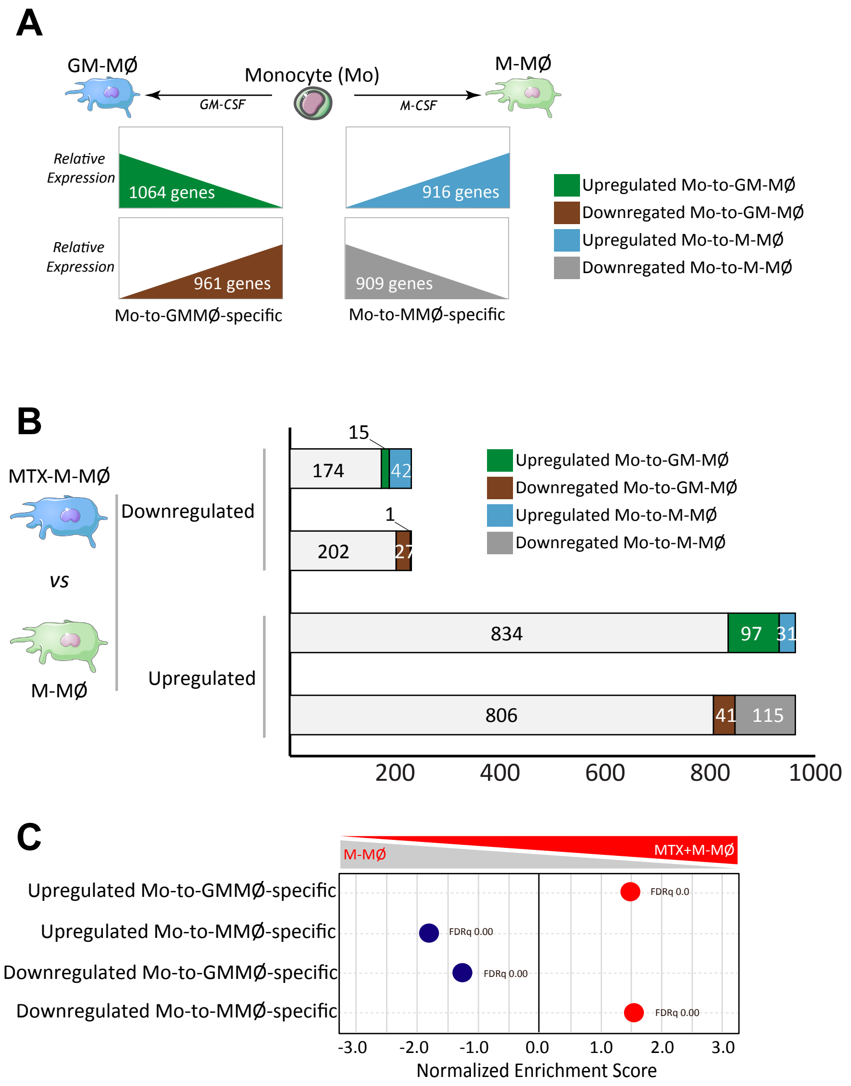

Supplementary Figure 2.- Expression of MTX-regulated genes along monocyte to macrophage differentiation (A) Schematic representation of the genes specifically upregulated or downregulated along the monocyte-to-M-MØ differentiation and monocyte-to-GM-MØ differentiation (log2FC>1; adj*p*<0.05) (GSE188278). **(B)** Comparison of downregulated or upregulated genes in M-MØ or MTX-M-MØ with the genes whose expression is upregulated or downregulated along the monocyte-to-M-MØ differentiation and monocyte-to-GM-MØ differentiation**. (C)** GSEA on the ranked comparison of the transcriptome of MTX-M-MØ versus untreated M-MØ, using the genes significantly modulated along the monocyte-to-M-MØ differentiation and monocyte-to-GM-MØ differentiation as data set. False discovery rate (FDRq) are indicated (red, positive enrichment; blue, negative enrichment). The genes within the leading edge of each GSEA are indicated in Supplementary Table 1.
