# Supplementary material for: GSK3β Inhibition Prevents Macrophage Reprogramming by High-Dose Methotrexate
Source: J Innate Immun. 2022 Nov 14;15(1):283–96. doi: 10.1159/000526622 (PMC10643894; doi:10.1159/000526622)
Supplement: Supplementary file 5 — Supplementary data [file jin-0015-0283-s05.pdf]

Supplementary Figure 3

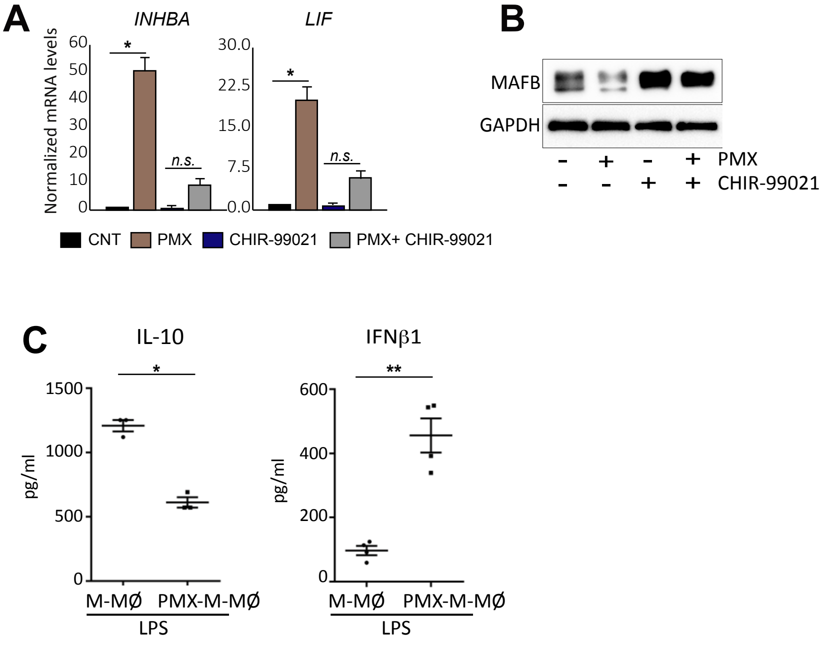

Supplementary Figure 3.- GSK3β inhibition modulates the expression of *INHBA*, *LIF* mRNA and MAFB in Pemetrexed (PMX) treated-M-MØ. (A) Gene expression of the indicated genes determined by qRT-PCR on M-MØ and PMX-M-MØ (d5). PMX and CHIR-99021 was added on day 5 and gene expression determined at day 7. Mean ± SEM of 4 independent donors are shown. Groups were compared by applying one-way ANOVA (with Tukey´s post hoc test, *$p<0.05$). **(B)** Immunoblot analysis of MAFB by M-MØ and PMX-M-MØ (d5) unexposed or exposed to CHIR-99021. GAPDH protein levels were determined as protein loading control. **(C)** Production of IL-10 and IFNβ by M-MØ and PMX-M-MØ challenged with LPS for 3h, as determined by ELISA. Mean ± SEM of 4 independent donors, each symbol represents a single donor (*, $p<0.05$, **, $p<0.01$, paired t-test).
